# Evaluation of the tracing effect of carbon nanoparticle and carbon nanoparticle-epirubicin suspension in axillary lymph node dissection for breast cancer treatment

**DOI:** 10.1186/s12957-016-0925-2

**Published:** 2016-06-22

**Authors:** Junze Du, Yongsong Zhang, Jia Ming, Jing Liu, Ling Zhong, Quankun Liang, Linjun Fan, Jun Jiang

**Affiliations:** Breast Surgery, Southwest Hospital, Third Military Medical University, Chongqing, 400038 China; Three Glands Surgery, the Second Affiliated Hospital, Chongqing Medical University, Chongqing, 400010 China

**Keywords:** Axillary lymph node dissection, Breast neoplasms, Carbon nanoparticles, Epirubicin

## Abstract

**Background:**

Carbon nanoparticle suspension, using smooth carbon particles at a diameter of 21 nm added with suspending agents, is a stable suspension of carbon pellets of 150 nm in diameter. It is obviously inclined to the lymphatic system. There were some studies reporting that carbon nanoparticles are considered as superior tracers for sentinel lymph nodes because of their stability and operational feasibility. However, there were few study concerns about the potential treatment effect including tracing and local chemotherapeutic effect of carbon nanoparticle-epirubicin suspension on breast cancer with axillary metastasis.

**Methods:**

In the current study, a randomized controlled analysis was performed to investigate the potential treatment effect of carbon nanoparticle-epirubicin suspension on breast cancer with axillary metastasis. A total of 90 breast cancer patients were randomly divided into three equal groups: control, tracer, and drug-load groups. The control group patients did not receive any lymphatic tracers, the tracer group patients were subcutaneously injected with 1 ml carbon nanoparticle suspension, and the drug-load group patients were injected with 3 ml carbon nanoparticle-epirubicin suspension at four separate sites around the areola 24 h before surgery. Modified radical mastectomy, endoscopic subcutaneous mammary resection plus axillary lymph node dissection, and immediate reconstruction with implants or breast-conserving surgery were performed.

**Results:**

The mean number of the dissected lymph nodes per patient was significantly higher in the tracer (21.3 ± 6.1) and drug-load (19.5 ± 3.7) groups than in the control group (16.7 ± 3.4) (*P* < 0.05). Most lymph nodes in the former two groups were stained black (75.7 and 73.3 %, respectively), but with no significant difference between the groups. Most metastatic lymph nodes were also stained black in the tracer group (68.6 %) and drug-load group (78.1 %) and with no significant difference between the groups (*P* = 0.198). Microscopic examination revealed that the carbon nanoparticles were localized around or among the cancer cell masses and residues of necrotized cancer cells surrounded by fibroblastic proliferation could be found within the stained lymph nodes in the drug-load group.

**Conclusions:**

The majority of axillary lymph nodes were stained black by the suspension of carbon nanoparticles, which helped identify the lymph nodes from the surrounding tissues and avoided aggressive axillary treatment. Thus, a combination therapy of carbon nanoparticles with epirubicin could play an important role in lymphatic chemotherapy without affecting tracing.

**Trial registration:**

ChiCTRTRC13003419

## Background

Breast cancer is the most common malignancy in women. Globally, its incidence is gradually increasing, and younger women have been diagnosed with this condition in recent years. Accumulated evidence indicates that axillary lymph node metastasis is an independent and crucial prognostic factor for breast cancer [1–4]. Historically, the axilla was treated with aggressive surgery, after the discovery of its function in regional lymph drainage and the possibility of harboring tumor deposits [5, 6]. However, over the last decades, a shift is being observed towards less invasive axillary treatment. With the introduction of the sentinel lymph node biopsy (SLNB) and axillary radiotherapy, axillary lymph node dissection (ALND) can be prevented in those patients with clinically node-negative breast cancer [7–10]. However, the current guidelines still recommend the performance of ALND in patients with clinically node-positive breast cancers. Therefore, in order to avoid aggressive axillary treatment, identifying the lymph nodes from the surrounding tissues is very important. It has been reported that carbon nanoparticles can be utilized as tracers for the complete elimination of axillary lymph nodes [11–14]. Further, a combination of carbon nanoparticles and chemotherapeutic drugs was used to eliminate cancer cells metastasized to lymph nodes such as the internal mammary lymph nodes, supraclavicular lymph nodes, and lymph nodes with skip metastasis, which cannot be accessed via conventional surgery [15, 16]. To our knowledge, there were few study concerns about the potential treatment effect including tracing and local chemotherapeutic effect of carbon nanoparticle-epirubicin suspension on breast cancer with axillary metastasis. In the current study, a randomized controlled analysis was performed to investigate the potential treatment effect of carbon nanoparticle-epirubicin suspension on breast cancer with axillary metastasis.

## Methods

### Preparation of carbon nanoparticle-epirubicin suspension

The carbon nanoparticle-epirubicin suspension was prepared as described previously [16, 17]. 0.10 mg of epirubicin hydrochloride for injection (Pharmorubicin RD, Pfizer Inc, Wuxi, China) was dissolved in 2 ml of normal saline and mixed thoroughly by moderate shaking. The epirubicin solution was then mixed with 1 ml suspension of carbon nanoparticles (1 ml:50 mg; Chongqing Lummy Pharmaceutical Co., Ltd., Chongqing, China), with shaking and vortexing for 5~10 min to allow complete binding of the nanoparticles and epirubicin. The final nanoparticle-epirubicin suspension was black.

### Inclusion and exclusion criteria

Patients in whom invasive breast cancer was confirmed by preoperative core-needle biopsy or the Mammotome^®^ biopsy system, those who showed no skin ulceration or pectoral muscle invasion, those with no obvious peau d’ orange on their breast or tumor surface, those with suspected axillary metastasis on preoperative clinical physical examination or color Doppler ultrasound examination, and those who provided signed informed consent were included in the study. Patients who had undergone excisional biopsy, those detected with fusion and fixed axillary lymph nodes before surgery, those in whom distant metastasis was detected before surgery, and those with a previous history of surgery on axillary lymph nodes were excluded from the study.

### Group division and tracing methods

**Fig. 1:**
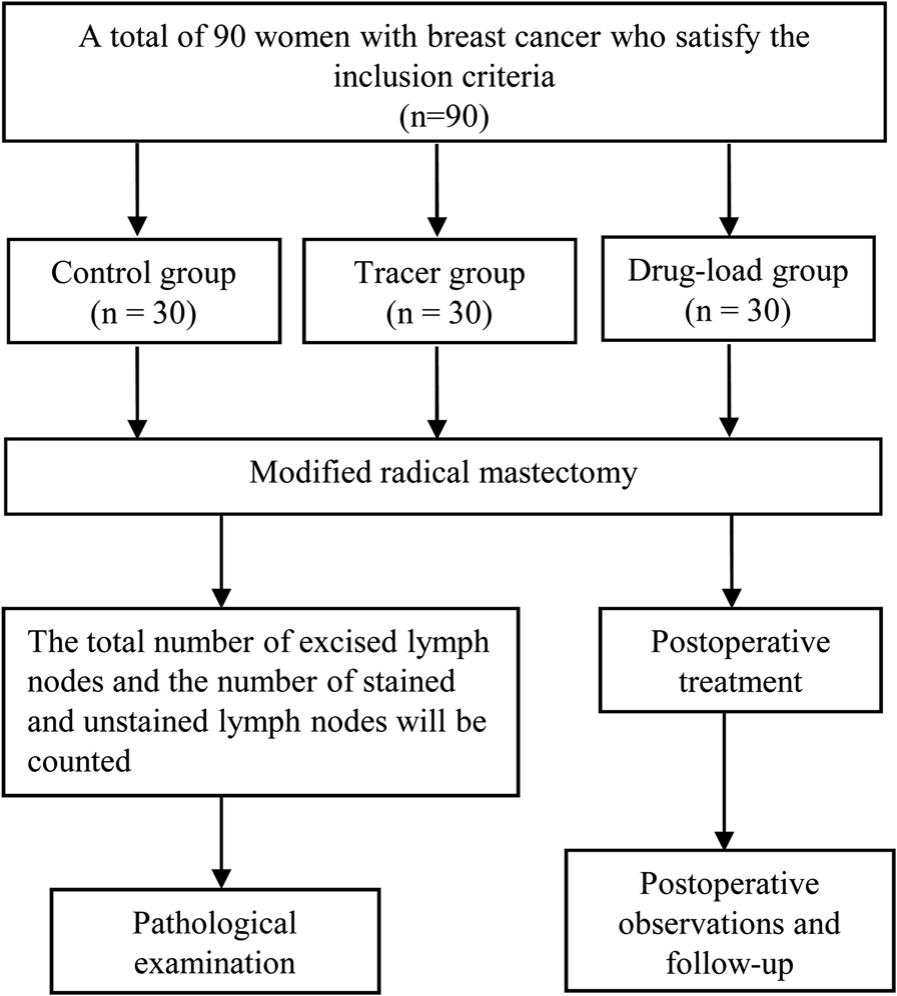
Flow diagram of the experiment

A total of 90 women with breast cancer aged 26–68 (mean, 47.0 ± 8.7) years who satisfied the inclusion criteria between February 2012 and September 2013 were enrolled in the current study. The breast tumor was located in the upper outer quadrant of the mammary gland in 65 of these patients, in the central area of the gland in 5 patients, in the lower outer quadrant in 8 patients, in the upper quadrant in 10 patients, and in the lower inner quadrant in 2 patients. The patients were divided into three groups using the random number method and single blind (Fig. 1). The patients belonging to the control group did not receive any kind of lymphatic tracers. Those belonging to the tracer and drug-load groups were subcutaneously injected with 1 and 3 ml suspensions of carbon nanoparticles and carbon nanoparticle-epirubicin, respectively, at four separate sites (0.25 ml for each site in the tracer group and 0.75 ml for each site in the drug-load group) around the areola 24 h before surgery. The injection site was lightly pressed with a cotton swab or gauze for a minute to prevent drug leakage. Any adverse reactions such as local pain, redness, fever, and allergy were recorded. ALND was then performed according to the literature [14]. There were three different surgeons who operated on the group, and the extent of axillary nodal dissection was levels I and II.

### Axillary lymph node examination and number count

During the surgery, the dissected lymph nodes were carefully isolated and counted from freshly removed tissues by specialized technical personnel. The total number of excised lymph nodes and the number of stained and unstained lymph nodes were counted and recorded for each patient. All lymph nodes and axillary tissues were subjected to pathological examination to clarify the metastatic condition.

### Neoadjuvant therapy

Patients receiving neoadjuvant therapy were eligible. There were 21 patients who received neoadjuvant therapy in the tracer group, 21 in the drug-load group, and 20 in the control group, totaling 62 patients (69 %) in the study. Chemotherapy regimens were TE-regime chemotherapy (intravenous injection of docetaxel at a dosage of 75 mg/m^2^ and epirubicin hydrochloride intravenous injection at a dosage of 80 mg/m^2^ for 14 days as 1 cycle) for 4 cycles.

### Postoperative treatment

Adjuvant chemotherapy was administered after surgery in accordance with the National Comprehensive Cancer Network (NCCN) guidelines, based on the histological type, lymph node metastasis, and pathological findings defined by the 2010 American Joint Committee on Cancer (AJCC) staging system for breast cancer. Whole-breast radiotherapy was performed for patients who had undergone breast-conserving surgery, while regional radiotherapy was performed for those who had more than four metastatic axillary lymph nodes. Endocrine therapy for a period of 5 years was recommended for patients whose estrogen receptor examination showed positive results. Molecular-targeted therapy was recommended for the human epidermal growth factor receptor 2 (HER-2)-positive patients.

### Postoperative observations and follow-up

Postoperative morbidity included infection, flap necrosis, edema of the upper arm, and shoulder dysfunction. The method used to measure upper arm edema is as follows: measure the affected arm circumference of the location 10 cm up and below the olecranon, then compare with the other side. In mild edema, the difference of the arm circumference of the two sides is <3 cm; in the moderate edema, the difference of the arm circumference of the two sides is approximately between 3 and 6 cm; in severe edema, the difference of the arm circumference of the two sides is >6 cm [18]. All the patients were followed up every 3 months in the outpatient department within the first year after surgery and subsequently every 6 months for review examination. The time and location of local recurrence or distant metastasis along with skin necrosis or systematic adverse effects due to the carbon nanoparticle-epirubicin suspension were recorded. The functions of vital organs such as the heart, liver, and kidney were examined at each follow-up. Any surgery-related adverse effects such as postoperative infection at the incision site, edema of the affected limb, skin flap necrosis, and subcutaneous fluid effusion along with other local and systematic adverse effects were also recorded.

### Statistical analysis

Data were analyzed using the Statistical Package for the Social Sciences Version 13.0 (SPSS Inc, Chicago, IL, USA). Measurement data were analyzed using the chi-square test, and quantitative data were presented as mean ± standard deviation. Univariate analysis was used for comparison among multiple groups, and the least significant difference test or Tamhane’s T2 test was used to compare two groups. *P* values were derived from two-tailed tests. *P* < 0.05 was considered statistically significant.

## Results

### Pathological examinations

A total of 63 patients underwent modified radical mastectomy, 24 underwent endoscopic subcutaneous mammary resection along with ALND and immediate reconstruction with implants, and 3 underwent breast-conserving surgery along with ALND. There were 22, 49, and 19 patients with breast cancer in the pathological grades of stage I, stage II, and stage III, respectively. Histological analysis showed that 87, 2, and 1 patients had infiltrating ductal carcinoma, mucinous carcinoma, and medullary carcinoma, respectively. The expressions of ER, PR, and HER-2 are shown in Table 1. There was no significant difference among the three groups in terms of the average age of the patients, the surgical approach, the pathological stage of cancer, pathological type, tumor location, hormone receptors, and HER-2 expression.

**Table 1:** Demographic information

Clinical pathological feature	Control (*n* = 30)	Tracer (*n* = 30)	Drug-load (*n* = 30)	Test value	*P*
Average age (year)	45.4 ± 7.8	47.1 ± 8.3	48.4 ± 9.8	0.885*	0.416
Tumor location				10.258^#^	0.247
Upper outer quadrant	18	21	26		
Lower outer quadrant	5	3	0		
Upper inner quadrant	3	4	3		
Lower inner quadrant	1	0	1		
Central area	3	2	0		
Clinical stage				2.018^#^	0.732
I	8	8	6		
II	18	15	16		
IIIA	4	7	8		
Pathological type				3.000^#^	0.558
Infiltrating ductal carcinoma	29	29	29		
Mucinous carcinoma	1	1	0		
Medullary carcinoma	0	0	1		
ER				1.348^#^	0.510
Positive	19	20	23		
Negative	11	10	7		
PR				0.098^#^	0.952
Positive	20	19	20		
Negative	10	11	10		
HER-2				4.229^#^	0.121
Positive**	3	8	3		
Negative^##^	27	22	27		
Surgical approach				4.488^#^	0.344
Modified radical mastectomy	20	25	18		
Endoscopic subcutaneous mammary resection + axillary lymph nodes dissection + reconstruction with implants	9	4	11		
Breast-conserving surgery + axillary lymph nodes dissection	1	1	1		

*ER* estrogen receptor, *PR* progesterone receptor, *HER-2* human epidermal growth factor receptor 2, *IHC* immunohistochemistry

*F value; #*χ* ^2^ value; **IHC (3+) in the first biopsy before surgery; ^##^IHC (0 ~ 2+) in the first biopsy before surgery

### Number of dissected lymph nodes and staining rate

A total of 502 (mean, 16.7 ± 3.4), 638 (mean, 21.3 ± 6.1), and 584 (mean, 19.5 ± 3.7) lymph nodes were dissected from the control group, tracer group, and drug-load group, respectively. Thus, the number of dissected lymph nodes was significantly greater in the drug-load and tracer groups than in the control group (*P* = 0.003 and 0.013), although there was no significant difference in the number of dissected lymph nodes between the former two groups (*P* = 0.433) (Table 2).

**Table 2:** Dissected nodes information

	Control	Tracer	Drug-load
Total number of nodes removed	502 (mean, 16.7 ± 3.4)	638 (mean, 21.3 ± 6.1)	584 (mean, 19.5 ± 3.7)
Total number of metastatic nodes	50	70	73
Total number of stained nodes	–	483	428
The number of metastatic stained nodes	–	48	57
The number of metastatic unstained nodes	–	22	16

**Fig. 2:**
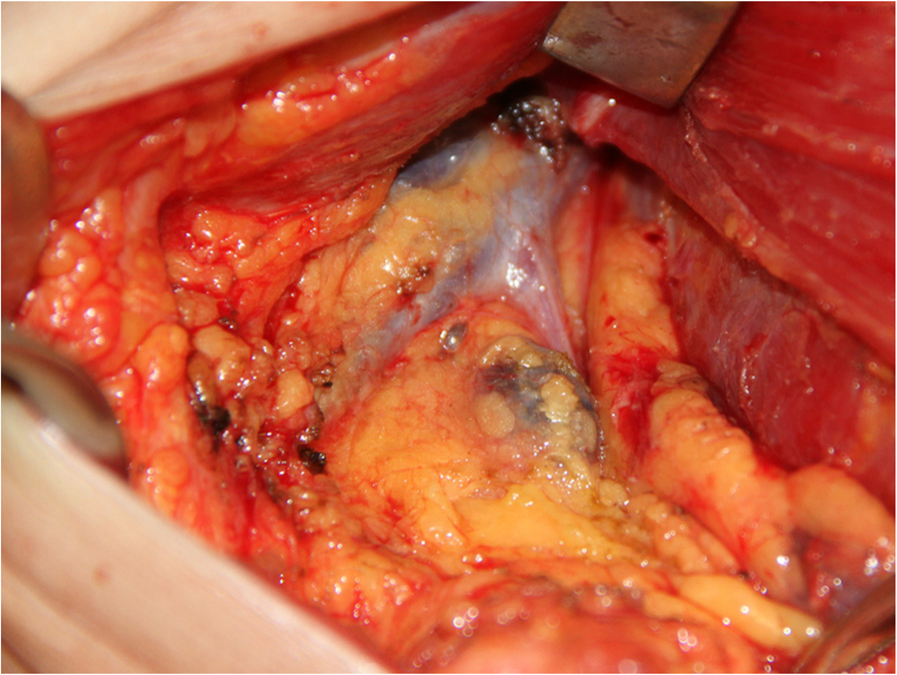
Stained lymph nodes during right axillary lymph node dissection 24 h after injection of carbon nanoparticles

**Fig. 3:**
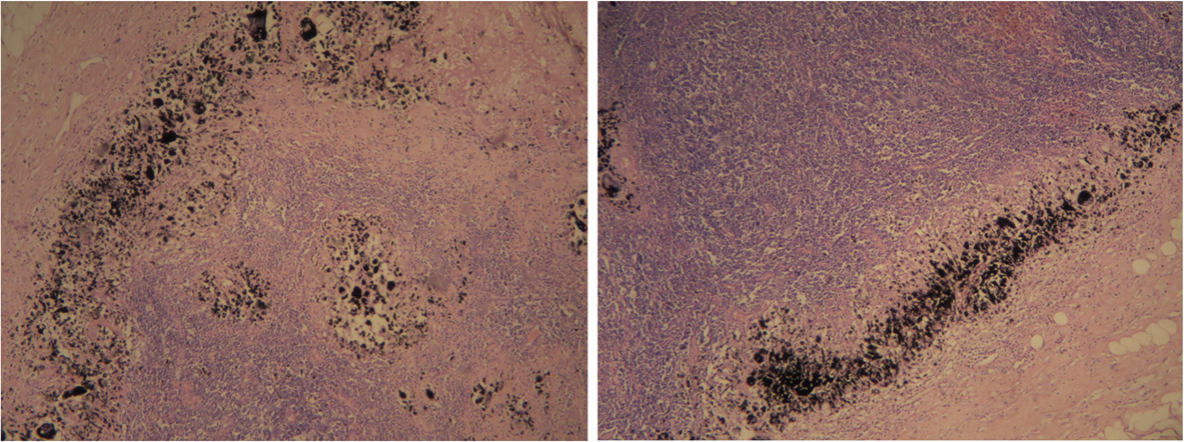
A large number of carbon nanoparticles localized in the nodal marginal sinus of stained lymph nodes from the tracer group (*left*) and drug-load group (*right*) (hematoxylin and eosin, ×40)

Majority of the lymph nodes were stained black by both carbon nanoparticle and nanoparticle-epirubicin suspension. These stained lymph nodes were much easier to identify during open surgery as well as under endoscopy (Fig. 2). The percentage of stained lymph nodes was 75.7 % (483/638) and 73.3 % (428/584) in the tracer group and drug-load group, respectively, and there was no significant difference with respect to the dye staining efficiency (*χ*^2^ = 0.939, *P* = 0.332) between the groups. A large amount of carbon nanoparticles that remained in the lymph nodes in both the tracer and drug-load groups was observed under the microscope (Fig. 3).

### Tracing and lymphatic chemotherapy effects

The number of dissected lymph nodes was 502, 638, and 584 in the control group, tracer group, and drug-load group, with 50, 70, and 73 metastases, respectively. Although more metastatic lymph nodes had been detected in the tracer group and drug-load group, there was no significant difference in the metastasis rate among the three groups (*χ*^2^ = 1.802, *P* = 0.406).

Of the 30 patients in the control group, 11 showed axillary lymph node metastasis. A total of 50 metastatic lymph nodes were excised from these patients. Of the 30 patients in the tracer group, 14 showed axillary lymph node metastasis. A total of 70 metastatic lymph nodes were excised from these patients, 48 of which were stained black (68.6 %). Of the 30 patients in the drug-load group, 14 showed axillary lymph node metastasis. A total of 73 metastatic lymph nodes were excised from these patients, 57 of which were stained black (78.1 %). The rate of staining of the metastatic lymph nodes did not significantly differ between the groups (*χ*^2^ = 1.657, *P* = 0.198).

A total of 568 non-metastatic lymph nodes were excised in the tracer group, 435 of which were stained black (76.6 %). A total of 511 non-metastatic lymph nodes were excised in the drug-load group, 371 of which were stained black (72.6 %). The rate of staining of the non-metastatic lymph nodes did not significantly differ between the groups (*χ*^2^ = 2.257, *P* = 0.133).

A total of 42 patients (70 %) received neoadjuvant therapy in the tracer group and drug-load group (neoadjuvant subgroup), and 18 patients (30 %) did not receive neoadjuvant therapy (no prior therapy subgroup). In the neoadjuvant subgroup, 649/884 (73.41 %) lymph nodes were stained black. In the no prior therapy subgroup, 263/338 (77.81 %) lymph nodes were stained black. The rate of staining of lymph nodes did not significantly differ between the neoadjuvant subgroup and the no prior therapy subgroup (*χ*^2^ = 2.494, *P* = 0.114). A total of 122 metastatic lymph nodes were excised in the neoadjuvant subgroup, 90 of which were stained black (73.77 %). A total of 21 metastatic lymph nodes were excised in the no prior therapy subgroup, 15 of which were stained black (71.42 %). There was no significant difference in the metastasis rate between the two groups (*χ*^2^ = 0.050, *P* = 0.822).

**Fig. 4:**
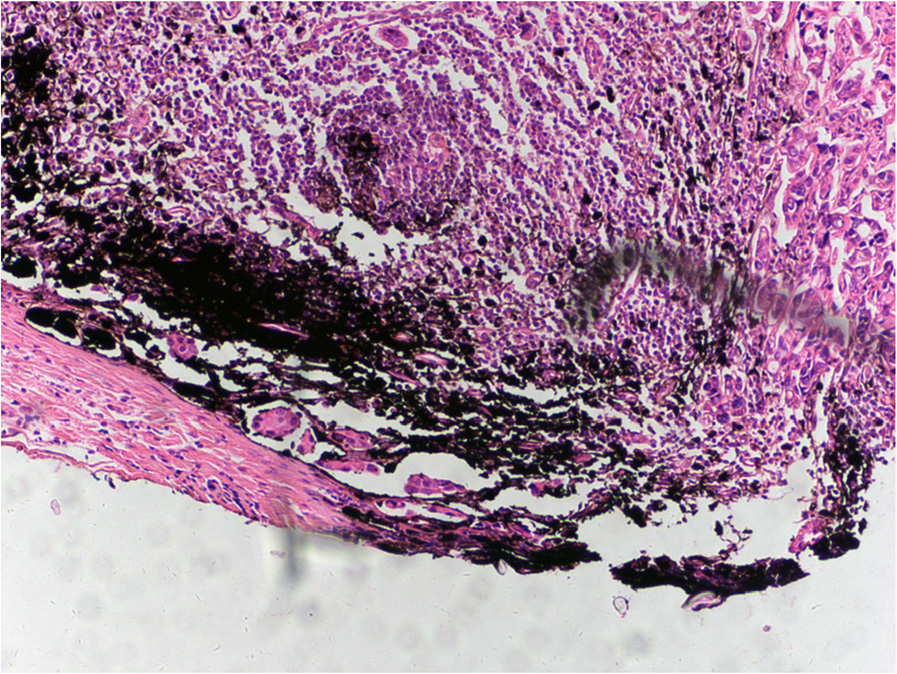
Metastatic lymph nodes stained with carbon nanoparticles in the tracer group and the presence of carbon particles around or between cancer cells (hematoxylin and eosin, ×100)

**Fig. 5:**
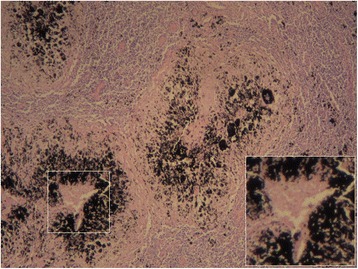
A large number of carbon nanoparticles localized in the nodal marginal sinus of stained lymph nodes from the drug-load group, with some cell necrosis observed (see the *right bottom corner*) (hematoxylin and eosin, ×40)

Microscopic examination revealed that the carbon nanoparticles were localized around or among the cancer cell masses of the stained lymph nodes (Fig. 4). Residues of necrotized cancer cells surrounded by fibroblastic proliferation could be found within the stained lymph nodes in the drug-load group (Fig. 5).

### Adverse effects of carbon nanoparticle-epirubicin suspension injection

Mild pain at the injection site was observed in the two groups; however, no treatment was required. No allergy, redness or swelling, necrosis or ulceration of the skin, or fever or lung injury, heart disease, and asthma were reported in any of the groups. Postoperative pathological examination showed no necrosis or inflammatory reaction, and the skin flap at the injection site was healing well without necrosis.

### Follow-up

The patients were followed up for a median duration of 28 (range, 20–39) months. Table 3 shows the incidence of treatment-related morbidity. During this period, no recurrent or distant metastasis or any obvious abnormality in the vital organs such as the heart, liver, and kidney was noted in any of the 90 patients.

**Table 3:** Postoperative morbidity information

Parameter	Control	Tracer	Drug-load	*P* value
Infection	1 (3.33 %)	0 (0 %)	1 (3.33 %)	0.600
Flap necrosis	3 (8.46 %)	1 (7.01 %)	0 (0 %)	0.160
Edema of upper arm	2 (6.67 %)	4 (13.33 %)	3 (10 %)	0.690
Shoulder dysfunction	0 (0 %)	0 (0 %)	0 (0 %)	

## Discussion

Carbon nanoparticles are novel lymphatic tracers with a good target efficiency and safety profile; no obvious cell toxicity or teratogenesis, adverse effect, or complication because of carbon nanoparticles has yet been reported [14, 16, 17, 19–24]. Carbon nanoparticles are activated carbon products processed by nanotechnology, which have a uniform diameter of 21 nm. In order to obtain a suspension, these nanoparticles are coupled with a suspending agent and saline, their final diameter being 150 nm. The gap size in the capillary endothelial junction ranges between 30 and 50 nm, with the complete basement membrane, whereas the gap size in the lymphatic capillary endothelial junction ranges between 100 and 500 nm, with loose connections and an incomplete basement membrane. Therefore, the carbon nanoparticles cannot enter into the blood vessels but enter the lymphatic vessels and undergo macrophage phagocytosis. The phagocytized particles aggregate and are retained in the lymph nodes, imparting a black color to the lymph nodes, thereby allowing in vivo staining of the draining lymph nodes around the tumor. Thus, carbon nanoparticles are considered as superior tracers for sentinel lymph nodes because of their stability and operational feasibility [25]. Our previous studies indicated that carbon nanoparticles are transferred to the next station of sentinel lymph nodes and thus achieve extensive coverage of lymph node staining. In the current study, the number of dissected lymph nodes was higher in both the tracer and drug-load groups than in the control group, and majority of the dissected lymph nodes were stained black, indicating the superior tracing effect of carbon nanoparticles, which help identify the lymph nodes from the surrounding tissues and avoided aggressive axillary treatment. Furthermore, it helps the pathologist identify more nodes in specimen processing.

Our findings also indicated that there was no significant difference in the rate of lymph node staining between the tracer group and drug-load group, which implied that the epirubicin-adsorbed carbon nanoparticles did not affect the staining property of the lymph nodes. Moreover, the carbon nanoparticles also stained majority of the metastatic lymph nodes in both groups, with staining rates of 68.6 and 78.1 % in the tracer group and drug-load group, respectively. Thus, carbon nanoparticles could improve the detection rate of metastatic lymph nodes and reduce the risk of missing their dissection during surgery.

Carbon nanoparticles possess a honeycomb-like structure and surface adsorption ability, which enable them as chemotherapeutic drug carriers to penetrate the lymph nodes and slowly release chemotherapeutics [16, 17, 26–28]. Previous studies in the VX2 rabbit breast cancer model in which the carbon nanoparticle-epirubicin suspension was injected every other day into the model for 6 days before operation found death of cancer cells in the metastatic lymph nodes [29]. In a clinical trial, breast cancer patients were injected with a suspension of activated carbon nanoparticles. This suspension served as an effective chemotherapeutic against axillary lymph nodes and elevated the concentration of chemotherapeutics within the lymph nodes [18]. Additionally, a homemade suspension of carbon nanoparticle-epirubicin was also used in breast cancer patients, and administration of this suspension 72 h before surgery increased the concentration of epirubicin in the axillary lymph nodes, thereby facilitating chemotherapy. In the current study, the carbon nanoparticle-epirubicin suspension was injected 24 h before surgery, which resulted in a moderate amount of cell necrosis and fibroblast proliferation within the stained lymph nodes. However, no necrosis was found in the tracer group, which implied that epirubicin was carried by carbon nanoparticles and transferred to the axillary lymph nodes via lymphatic drainage channels, thereby fulfilling its role in lymph node-targeted chemotherapy. A better therapeutic effect might be achieved with earlier dosing, an increased dose, and increased dosing frequency, and this should be verified in future clinical studies.

Epirubicin is a commonly used cytotoxic anticancer drug, and it is the first-line chemotherapeutic against breast cancer. However, epirubicin alone is not recommended for injection, as it could lead to skin ulcers and necrosis in case of any leakage in the extravascular tissue. The adsorption experiment of carbon nanoparticles indicated that the epirubicin (2 ml) adsorption rate of 1 ml (50 mg) carbon nanoparticles could reach 85.6 and 85.7 % at epirubicin concentrations of 5 and 10 mg/l, respectively [30]. Liang et al. worked on nude mouse models and observed that mitomycin C could be maintained on nanoparticles for 24 h before being completely released in vivo [31]. Thus, the adsorption property of carbon nanoparticles enables the localization of a high concentration of chemotherapeutics within targeted lymph nodes and helps avoiding the adverse effect of skin degeneration and necrosis after local injection.

The safety profile of the carbon nanoparticle-epirubicin suspension was determined through an experiment conducted in rats, in which no skin necrosis or ulceration was observed at the injection site 1 week after injection and the local adverse reaction was significantly reduced [32]. In the current study as well, subcutaneous injection of the carbon nanoparticle-epirubicin suspension around the areola of the patients did not result in any adverse reactions such as skin allergy, redness, swelling, ulceration, degeneration, or fever before surgery and during the follow-up period of 20–39 months. Postoperative histological examination revealed no necrosis or inflammatory reactions, and the skin flap was growing well without necrosis. Similarly, no obvious edema of the affected upper limbs or subcutaneous effusion was observed in any of the patients. Thus, our results confirmed the safety of the carbon nanoparticle-epirubicin suspension.

## Conclusions

In summary, our findings indicate that the carbon nanoparticle-epirubicin suspension is a safe and effective lymph node tracer and lymphatic chemotherapeutic for axillary lymph nodes in breast cancer treatment. Most of the axillary lymph nodes were stained black by the carbon nanoparticle suspension, which enabled their easy identification from the surrounding tissues and in turn their complete dissection. The metastatic lymph nodes did not seem to affect the tracing properties of carbon nanoparticles. Therefore, carbon nanoparticles adsorbed with epirubicin could act as lymphatic chemotherapeutics without sacrificing their tracing effect. It may be helpful for the treatment of breast cancer with axillary metastasis.

## Abbreviations

ALND, axillary lymph node dissection; BCS, breast-conserving surgery; ER, estrogen receptor; HER-2, epidermal growth factor receptor 2; NACT, neoadjuvant chemotherapy; PR, progesterone receptor; SLNB, sentinel lymph node biopsy

## References

[1] Rugina VG, Mihalcea D, Pricop F (2011). The lymph nodes status-prognostic factor in breast cancer. Rev Med Chir Soc Med Nat Iasi.

[2] Stankov A, Bargallo-Rocha JE, Silvio AN, Ramirez MT, Stankova-Ninova K, Meneses-Garcia A (2012). Prognostic factors and recurrence in breast cancer: experience at the national cancer institute of Mexico. ISRN Oncol.

[3] Georgescu R, Coros MF, Stolnicu S, Podeanu D, Sorlea S, Rosca A (2012). Prognostic factors in breast cancer. Rev Med Chir Soc Med Nat Iasi.

[4] He M, Tang LC, Yu KD, Cao AY, Shen ZZ, Shao ZM (2012). Treatment outcomes and unfavorable prognostic factors in patients with occult breast cancer. Eur J Surg Oncol (EJSO).

[5] Halsted WSI (1898). A clinical and histological study of certain adenocarcinomata of the breast: and a brief consideration of the supraclavicular operation and of the results of operations for cancer of the breast from 1889 to 1898 at the Johns Hopkins Hospital. Ann Surg.

[6] Suami H, Pan WR, Taylor GI (2009). Historical review of breast lymphatic studies. Clin Anat.

[7] Giuliano AE, Kirgan DM, Guenther JM, Morton DL (1994). Lymphatic mapping and sentinel lymphadenectomy for breast cancer. Ann Surg.

[8] Kim T, Giuliano AE, Lyman GH (2006). Lymphatic mapping and sentinel lymph node biopsy in early-stage breast carcinoma: a metaanalysis. Cancer.

[9] Giuliano AE, McCall L, Beitsch P (2010). Locoregional recurrence after sentinel lymph node dissection with or without axillary dissection in patients with sentinel lymph node metastases: the American College of Surgeons Oncology Group Z0011 randomized trial. Ann Surg.

[10] Donker M, van Tienhoven G, Straver ME (2014). Radiotherapy or surgery of the axilla after a positive sentinel node in breast cancer (EORTC 10981–22023 AMAROS): a randomised, multicentre, open-label, phase 3 non-inferiority trial. Lancet Oncol.

[11] Fan L, Strasser-Weippl K, Li JJ, St LJ, Finkelstein DM, Yu KD (2014). Breast cancer in China. Lancet Oncol.

[12] Sawai K, Hagiwara A, Shimotsuma M, Sakakibara T, Imanishi T, Takemoto Y (1996). Rationale of lymph node dissection for breast cancer—from the viewpoint of analysis of axillary lymphatic flow using activated carbon particle CH40. Gan To Kagaku Ryoho.

[13] Yokota T, Saito T, Narushima Y, Iwamoto K, Iizuka M, Hagiwara A (2000). Lymph-node staining with activated carbon CH40: a new method for axillary lymph-node dissection in breast cancer. Can J Surg.

[14] Fan LJ, Zhong L, Guo DY, He QQ, Jiang J (2010). Effect and safety of carbon nanoparticles dyeing in axillary lymph node dissection of breast cancer. Chin J Breast Dis (Electronic Edition).

[15] Chen JH, Wang L, Yao Q, Ling R, Li K, Wang H (2004). Drug concentrations in axillary lymph nodes after lymphatic chemotherapy on patients with breast cancer. Breast Cancer Res.

[16] Yang Q, Wang XD, Chen J, Tian CX, Li HJ, Chen YJ (2012). A clinical study on regional lymphatic chemotherapy using an activated carbon nanoparticle-epirubicin in patients with breast cancer. Tumour Biol.

[17] Guo F, Mao X, Wang J, Luo F, Wang Z (2011). Gemcitabine adsorbed onto carbon particles increases drug concentrations at the injection site and in the regional lymph nodes in an animal experiment and a clinical study. J Int Med Res.

[18] Jia WU, Yaqun ZHU, Ye TIAN, Nan JIANG (2013). Research and risk factors associated with the development of breast cancer-related lymphedema after irradiation. Chin Clin Oncol.

[19] Huczko A, Lange H, Calko E (1999). Short communication: fullerenes: experimental evidence for a null risk of skin irritation and allergy. Fuller Sci Technol.

[20] VanTongeren MJ, Kromhout H, Gardiner K (2000). Trends in levels of inhalable dust exposure, exceedance and overexposure in the European carbon black manufacturing industry. Ann Occup Hyg.

[21] Ito T, Hagiwara A, Takagi T, Fujiyama J, Sonoyama Y, Shimomura K (2003). Local administration of methotrexate bound to activated carbon particles (MTX-CH) for treating cancers in mice. Anticancer Res.

[22] Warheit DB, Laurence BR, Reed KL, Roach DH, Reynolds GA, Webb TR (2004). Comparative pulmonary toxicity assessment of single-wall carbon nanotubes in rats. Toxicol Sci.

[23] Fiorito S, Serafino A, Andreola F, Togna A, Togna G (2006). Toxicity and biocompatibility of carbon nanoparticles. J Nanosci Nanotechnol.

[24] Cai HK, He HF, Tian W, Zhou MQ, Hu Y, Deng YC (2012). Colorectal cancer lymph node staining by activated carbon nanoparticles suspension in vivo or methylene blue in vitro. World J Gastroenterol.

[25] Ge J, Yan B, Cao XC (2011). Comparison of sentinel lymph node detection by methylene blue and carbon nanoparticle suspension injection in early breast cancer. Zhonghua Zhong Liu Za Zhi.

[26] Hagiwara A, Hirata Y, Takahashi T (1998). A pilot study of fiberscopy-guided local injection of anti-cancer drugs bound to carbon particles for control of rectal cancer. Anticancer Drugs.

[27] Hagiwara A, Torii T, Sawai K, Sakakura C, Shirasu M, Ohgaki M (2000). Local injection of anti-cancer drugs bound to carbon particles for early gastric cancer—a pilot study. Hepatogastroenterology.

[28] Nakase Y, Hagiwara A, Kin S, Fukuda K, Ito T, Takagi T (2004). Intratumoral administration of methotrexate bound to activated carbon particles: antitumor effectiveness against human colon carcinoma xenografts and acute toxicity in mice. J Pharmacol Exp Ther.

[29] Chen JH, Ling R, Wang L, Yao Q, Li KZ, WANG Z (2005). Local administration of activated carbon-adsorbed mitomycin C in treatment of breast cancer model with metastasized axillary lymph nodes. Chin J Cancer Prev Treatment.

[30] Yang Q, Chen J, Tian CX, Wang R, Fan XJ, Lv Q (2012). The property with adsorption and slow release of carbon nanoparticles suspension injection for epirubicin solution in vitro. Chin J Bases Clin Gen Surg.

[31] Liang H, Tang HW, Hao XS, Sun H, Li W (2005). Pharmacokinetic study of intraperitoneal chemotherapy with mitomycin C bound to activated carbon particles. Chin J Oncol.

[32] Yang Q, Chen J, Tian CX, Fan XJ, Wang R, Lv Q (2012). Study on local application of epirubicin-absorbing activated carbon nanoparticles. Chin J Exp Surg.

